# Blood Acylated Ghrelin Concentrations in Healthy Term Newborns: A Prospective Cohort Study

**DOI:** 10.1155/2022/9317425

**Published:** 2022-02-24

**Authors:** Neha Parveen, Ayesha Ahmad, Syed Manazir Ali, Shagufta Moin, Nasreen Noor

**Affiliations:** ^1^Department of Pediatrics, JN Medical College and Hospital, Faculty of Medicine, Aligarh Muslim University, Uttar Pradesh, Aligarh 202001, India; ^2^Department of Biochemistry, JN Medical College and Hospital, Faculty of Medicine, Aligarh Muslim University, Uttar Pradesh, Aligarh 202001, India; ^3^Department of Obstetrics and Gynaecology, JN Medical College and Hospital, Faculty of Medicine, Aligarh Muslim University, Uttar Pradesh, Aligarh 202001, India

## Abstract

**Objective:**

The effect of ghrelin, a growth hormone (GH) secretagogue on growth of neonates, has been studied in the past, but not fully clarified. We aimed to investigate the relationship between ghrelin and growth parameters at birth and at the age of three months in healthy term infants. *Methodology*. This was a prospective observational study carried out in a tertiary care hospital. Eighty-four infants born at gestational ages between 37 and 42 weeks and classified as term small for gestational age (SGA) and appropriate for gestational age (AGA) were included in the study. Estimation of acylated ghrelin (AG) concentrations was done in the cord blood at birth and in venous blood at the age of 3 months in all the infants. The correlation between AG concentrations and growth parameters at birth and at 3 months was studied.

**Results:**

AG concentrations were significantly higher in SGA (236.16 ± 152.4 pg/ml) than AGA neonates (59.45 ± 20.95 pg/ml) at birth. Concentrations were observed to be negatively correlated with birth weight (*r* = −0.34, *p* value 0.03), birth length, and head circumference (*r* = −0.509 and -0.376, respectively) in SGA neonates. However, at 3 months, AG concentrations did not correlate with changes in anthropometric parameters in both the groups.

**Conclusion:**

Cord acylated ghrelin concentrations are higher in SGA neonates, and the concentrations are inversely proportional to the birth weight. Hence, its role as a surrogate marker for intrauterine nutrition can be suggested. However, its concentrations do not correlate with anthropometric parameters in early postnatal growth, suggesting it may not have a direct role in postnatal growth.

## 1. Introduction

Small for gestational age (SGA) infants are defined as neonates with birth weight for gestational age below the 10th centile as per gender-specific reference population. SGA infants are at greater risk for perinatal morbidity, persistent short stature, and metabolic alterations like diabetes, obesity, and cardiovascular disorders later in life [1]. With a prevalence of 34%, India stands among the top three countries with the highest number of SGA births [2]. Most of SGA infants show catch-up growth during the initial years with almost 90% achieving the age-appropriate height by 2 years [3, 4]. SGA infants have reduced intrauterine growth due to various maternal, fetal, and placental factors [5, 6]. Numerous factors predicting the postnatal catch-up growth have been studied [7, 8]. Ghrelin, being a GH secretagogue, has also been a topic of interest for its effect on postnatal catch-up growth.

Ghrelin, an acylated 28 amino acid peptide, was first discovered in the stomach of rats and has orexigenic and GH secretagogue properties [9]. In humans, it is secreted primarily by the stomach and exerts its effects on growth through stimulation of growth hormone secretagogue receptors. High concentrations of ghrelin gene expression have been detected in fetal pancreas suggesting it to be a major source of fetal ghrelin [10, 11]. During fetal life, ghrelin is secreted from the pituitary, lung, and placenta and concentrations are detected in fetal circulation as early as 20 weeks of intrauterine life [12]. Apart from GH secretion, it has been shown to stimulate appetite and induce adiposity through its hypothalamic neuropeptide activity [13]. It also plays an important role in the maintenance of chronic nutritional status and glucose homeostasis [14]. In an experimental study, human subjects who were given intravenous ghrelin reported increased ratings of hunger on a visual analogue scale [15]. With the assumption that umbilical cord ghrelin concentrations indicate its concentrations during intrauterine life, studies have explored the potential role of ghrelin in adaptation to chronic intrauterine malnutrition.

Acylated form of ghrelin was considered the active form of ghrelin [16] but a growing body of literature has profiled the complex identities and interactions between deacylated and acylated forms of ghrelin [17–19].

Birth weight being the most sensitive indicator of neonatal adiposity can be used as a surrogate marker of intrauterine growth, and its correlation with cord ghrelin concentrations can serve as a reliable marker of intrauterine growth and subsequent postnatal catch-up growth. As the prevalence of SGA in India is higher than that in the western population, the aetiologies are different considering differences in genetic and environmental factors [20]. With paucity of data exploring the role of ghrelin in catch-up growth SGA infants in a developing country, this study was planned to estimate the concentrations of acylated ghrelin in cord blood, its relation to birth anthropometry, and effects on postnatal growth.

## 2. Materials and Methods

### 2.1. Study Setting and Design

This was a single-centre prospective cohort study conducted in the Department of Pediatrics in a tertiary care setting in India. Ethical clearance was sought from the Hospital Ethics Committee (D. No. 1015/FM).

### 2.2. Study Participants

Term neonates born to healthy mothers in the hospital between October 2017 and October 2019 were considered for inclusion in the study.

Sample size was calculated using Cochran's formula 4*pq*/*d*2, where *p* is the prevalence, *q* is 1 − *p*, and *d* is the deviance (5-20% of *p*), *p* = 75, *q* = 25, and *d* = 20% of 75 = 15, 4 × 75 × 25/225 = 33.3 (10% for attrition and rounded off to 40 in each group).

**Figure 1:**
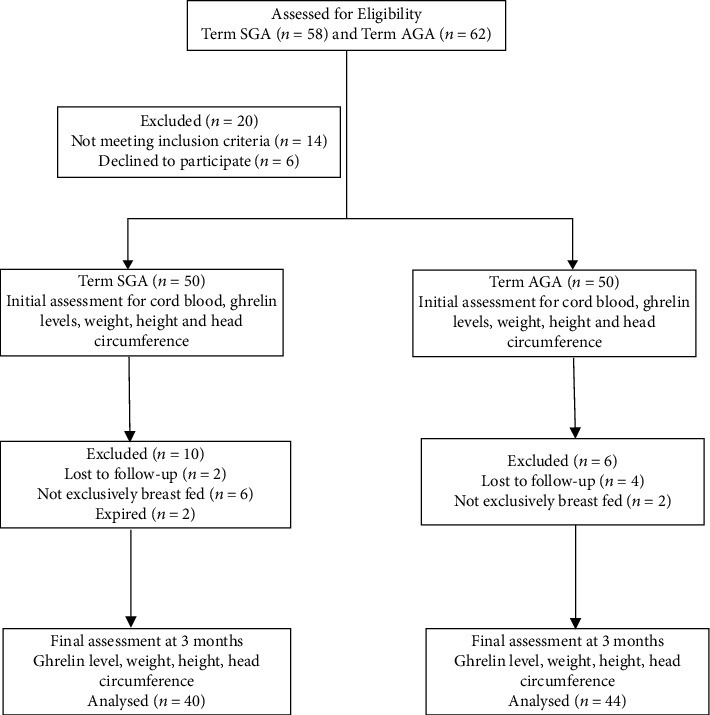
Flow chart of patients in the study.

Eighty-four infants—40 SGA and 44 AGA—were analysed at the end of study (Figure 1).

After informed consent was obtained from the parents, enrolled neonates were categorised as SGA with birth weight < 10th centile and AGA with birth weight between 10th and 90th centile as per the national growth standard charts [21]. Preterm infants, multiple gestation, infants with Apgar score < 8, congenital malformation, and those with maternal history of hypertension, smoking, and any chronic systemic disorders were excluded from the study. All the neonates were exclusively breastfed until the completion of the study.

### 2.3. Data Collection

Samples (5 ml) from the cord blood of neonates were collected in EDTA tubes, labelled appropriately, centrifuged, and treated with 1/10 N HCl. Thereafter, samples were stored at -70 degrees Celsius till the time of analysis. Estimation of ghrelin concentration was done by using Human Acylated Ghrelin Express ELISA Kit (Fine Test®, EH2601, Wuhan Fine Biological Technology Co., Ltd, Hubei, China) using the ELISA (A Microplate Reader, Alere®, AM-2000, Massachusetts, USA) method. A detailed maternal history, gestational age, and neonatal examination including anthropometric parameters were recorded in neonatal case records.

Naked birth weight was taken on an electronic weighing scale (GTET; Gold Tech®, New Delhi, India) with an accuracy of ±5 g. Length was measured using an infantometer (Narang Medical Limited, NET®, New Delhi, India) with a precision of ±0.1 cm. Head circumference was measured between the supraorbital ridge and the occipital protuberance using a nonstretchable tape. The average of three readings was recorded for all the parameters. At three months of age, anthropometric parameters were recorded by the same observer and venous samples for evaluation of acylated ghrelin concentrations were collected in the morning utilising the same procedure.

### 2.4. Statistical Analysis

All the data was expressed as mean ± SD. Correlation between different parameters was determined using Pearson's correlation test. The differences in variables between groups were compared using Student's *t*–test and categorical variables with the chi-square test. Statistical significance was set at *p* value < 0.05. All the statistical analyses were performed using version 20 of SPSS (2019).

## 3. Results

### 3.1. Anthropometric Parameters in SGA and AGA Infants at Birth and at Postnatal Age of 3 Months

Table 1 shows the findings of the infants at birth and at 3 months of age and the changes in the parameters over time. Gestational age was comparable in SGA and AGA groups; however, birth weight and length in SGA infants were lower than those in AGA infants because of the classification criteria.

At 3 months, SGA infants had significant weight gain (3.17 ± +0.17 kg), increase in length (8.32 ± 0.89 cm) and head circumference (5.97 ± 0.51). AGA infants also demonstrated significant gains in weight (3.13 ± 0.24 kg), length (7.55 ± 0.72 cm), and head circumference (5.23 ± 0.72). SGA infants had significant increases in length and head circumference compared to AGA infants. Though there were no significant differences in weight gain between SGA and AGA infants in absolute values, the percent gain in weight was observed to be significantly higher in SGA infants as compared to AGA infants (147.4% vs. 106.82%).

### 3.2. Acylated Ghrelin Concentrations in AGA and SGA Neonates at Birth and at Postnatal Age of 3 Months

SGA infants had significantly higher cord AG than AGA infants (236.16 ± 152.40 vs. 59.45 ± 20.95 pg/ml) at birth and at 3 months (652.32 ± 171.24 pg/ml vs. 361.36 ± 83.18). Changes in ghrelin levels were significantly higher in SGA infants than in AGA infants (418.66 ± 132.96 vs. 301.90 ± 89.89 pg/ml).

### 3.3. Correlation between Acylated Ghrelin Concentrations and Anthropometric Parameters in the Two Groups

Acylated ghrelin level was negatively correlated with birth weight (*r* = −0.34, *p* value 0.03), birth length, and head circumference (*r* = −0.509 and -0.376, respectively) in SGA neonates (Table 2).

However, at 3 months, AG concentration did not correlate with the changes in anthropometric parameters in both the groups.

### 3.4. Acylated Ghrelin Concentration and Gender

There were no significant differences in ghrelin concentrations among male and female infants of either group at birth and at 3 months (Table 3).

## 4. Discussion

In our study, the baseline cord AG concentrations were significantly higher in the SGA group. Similar observation was put forth by other studies with higher cord ghrelin in SGA as well as preterm infants [22–27]. However, most of these studies have measured total ghrelin concentrations rather than the acylated form which is the major bioactive form mediating effects on GH receptors [16]. In a study by Broglio et al., deacylated ghrelin was observed to have an antagonistic effect on glucose and insulin secretion with no effect on growth hormone or ACTH response [18]. In a study on Mexican newborns, researchers did not find any significant differences in acylated ghrelin concentrations between SGA and AGA infants [28]. The weight at birth is a reliable surrogate marker for intrauterine growth; this observation of higher AG concentrations in lighter infants suggests a potential role of ghrelin in the adaptation to chronic intrauterine malnutrition. The origin of ghrelin being fetal rather than placental has been demonstrated by Cortelazzi et al. [12].

At 3 months, there was a significant gain in length and head circumference in SGA neonates. There was no difference in weight gain between the two groups but the SGA group demonstrated a higher intragroup weight gain. Mean change in serum ghrelin concentrations was also observed to be significantly higher in SGA infants than in AGA infants. Our findings are in accordance with results in other similar studies [22–27, 29].

### 4.1. Correlation between Ghrelin Concentrations and Anthropometric Parameters at Birth and 3 Months

The exact mechanism by which ghrelin affects the weight gain in early infancy in humans is unclear, but a simple explanation would be that lower ghrelin concentrations are associated with poor appetite, resulting in lower nutritional intake. The effect of ghrelin on growth during the perinatal period is different from that on adolescents and adults. During the initial 2 years of life, concentrations of ghrelin are high, with progressive decline during childhood and adolescence [30, 31]. This suggests the possible role of ghrelin in catch-up growth in infants. In older children and adults, ghrelin concentrations are inversely related to weight and body mass index, with the concentrations being high in anorexia and low in obesity [32, 33]. Low cord ghrelin concentrations have been associated with poor weight gain at 3 months of age [34]. In a study by Iñiguez et al., infants who showed poor catch-up growth showed a larger decline in ghrelin concentrations after intravenous glucose administration [35]. However, in our study, though the AG concentrations increased more in SGA infants than AGA at 3 months, we did not find any significant correlation between AG and gain in weight and length at 3 months. This was in accordance with the findings by other researchers demonstrating no role of ghrelin in catch-up growth in SGA infants and even twins [36–38]. Sun et al. [39] demonstrated normal growth, bone density, and adiposity in mice who were ghrelin gene depleted. Our study supports the previous studies demonstrating increasing ghrelin concentrations in early infancy but suggests that ghrelin might not have a direct role in growth in early infancy. Therefore, synthetic ghrelin analogues might not have broad utility as growth-stimulating agents in SGA infants with poor postnatal growth.

### 4.2. Ghrelin Concentrations and Gender

This study showed no significant gender differences in AG concentrations in both groups at birth and at 3 months of age. This was consistent with other studies which have found either no gender differences or higher ghrelin concentrations among SGA girls citing a low birth weight in the females as the reason [23, 26, 30].

The major limitation of our study is that we have not measured growth hormone and insulin-like growth factor-1 concentrations which are the main mediators for the action of ghrelin. Another limitation is short-term follow-up of infants for 3 months only. As the ghrelin concentrations show a declining trend with age [36], a long-term follow-up can give a better picture about the effect of ghrelin in catch-up growth in SGA infants.

## 5. Conclusion

Cord acylated ghrelin concentrations are higher in SGA neonates, and the concentrations are inversely proportional to the birth weight. Hence, its role as a surrogate marker for intrauterine nutrition can be suggested. However, its concentrations do not correlate with anthropometric parameters in early postnatal growth suggesting it may not have a direct role in postnatal growth. Further research with a longer follow-up is needed to explore the role of ghrelin and other growth hormone secretagogues in catch-up growth of SGA infants.

## Figures and Tables

**Table 1:** Baseline anthropometry and acylated ghrelin concentrations in the study participants at birth and 3 months and changes in parameters.

	SGA (*n* = 40)	AGA (*n* = 44)	*t*	*p* value
Birth				
Gestational age (weeks)	37.84 ± 0.9	37.7 ± 0.8	0.455	0.324
Male/female	17/23	21/23		0.631
Weight (kg)	2.15 ± 0.25	2.93 ± 0.25	-13.85	<0.001
Length (cm)	44.84 ± 1.78	48.39 ± 1.10	-11.09	<0.001
Head circumference (cm)	31.81 ± 1.43	33.97 ± 0.82	-8.5	<0.001
Acylated ghrelin (pg/ml)	236.16 ± 152.40	59.45 ± 20.95	7.6	<0.001

3 months				
Weight (kg)	5.33 ± 0.30	6.07 ± 0.37	-9.86	<0.001
Length (cm)	53.17 ± 2.08	55.98 ± 1.40	-7.29	<0.001
Head circumference (cm)	37.76 ± 1.32	39.20 ± 0.85	-5.97	<0.001
Acylated ghrelin (pg/ml)	652.32 ± 171.24	361.36 ± 83.18	10.04	<0.001

Changes in parameters				
Weight gain (kg)	3.17 ± 0.17	3.13 ± 0.24	0.97	0.330
Weight gain %	147.4	106.82	-11.3	<0.001
Length gain (cm)	8.32 ± 0.89	7.55 ± 0.72	4.38	<0.001
Head circumference gain (cm)	5.97 ± 0.51	5.22 ± 0.73	5.28	<0.001
Acylated ghrelin (pg/ml)	418.66 ± 132.96	301.90 ± 89.89	4.75	<0.001

**Table 2:** Correlation between acylated ghrelin concentrations and anthropometric parameters at birth and at 3 months.

	SGA		AGA	
	*r*	*p* value	*r*	*p* value
At birth				
Weight (kg)	-0.341	0.030	0.175	0.256
Length (cm)	-0.509	0.001	0.218	0.155
Head circumference (cm)	-0.376	0.010	0.440	0.003

At 3 months				
Change in weight (kg)	-0.119	0.465	-0.075	0.630
Change in length (cm)	-0.020	0.903	-0.084	0.589
Change in head circumference (cm)	-0.033	0.839	0.002	0.990

**Table 3:** Effect of gender on acylated ghrelin concentrations in term SGA and AGA infants.

	SGA			AGA		
	Male (*n* = 17)	Female (*n* = 23)	*p* value	Male (*n* = 21)	Female (*n* = 23)	*p* value
Cord acylated ghrelin (pg/ml)	140.27 ± 120.79	178.971 ± 60.69	0.220	57.66 ± 23.44	61.08 ± 18.77	0.590
Serum acylated ghrelin at 3 months (pg/ml)	494.99 ± 144.70	521.37 ± 225.63	0.532	386.64 ± 65.51	338.27 ± 91.96	0.051

## Data Availability

The data used to support the findings of this study are available from the corresponding author upon request.
